# Portable HEPA air filtration effects on particulate matter, radioactivity, and chemical constituents in occupied homes

**DOI:** 10.1016/j.envint.2026.110528

**Published:** 2026-09-19

**Authors:** Choong-Min Kang, Joy Lawrence, Shaodan Huang, Jennifer Kang, Jun Young Yoo, Chan Jung Park, Mary B. Rice, Wanda Phipatanakul, Eric Garshick, Petros Koutrakis

**Affiliations:** a Department of Environmental Health, Harvard T.H. Chan School of Public Health, Boston, MA 02215, USA; b Department of Occupational and Environmental Health Sciences, Peking University, China; c Research Division, Coway R&D Center, Seoul 08826, South Korea; d Clinical Research Center, Boston Children’s Hospital Asthma, Allergy & Immunology, Dermatology & Rheumatology, Harvard Medical School, Boston, MA 02115, USA; e Pulmonary, Allergy, Sleep, and Critical Care Medicine Section, Medical Service, VA Boston Healthcare System; Harvard Medical School, Boston, MA 02130, USA

**Keywords:** HEPA intervention, Indoor particulate matter, Particle radioactivity, Reduced health risks

## Abstract

Although in-home air cleaners are known to reduce particulate matter (PM) concentrations, their effects on PM chemical constituents and particle-bound radioactivity (PR) under real-world residential conditions remain poorly characterized. We evaluated the effects of a portable high-efficiency particulate air (HEPA) cleaner on particle mass, chemical composition, and particle-bound radioactivity in occupied homes. We collected 86 indoor samples from 19 homes in the Boston metropolitan area between June 2017 and March 2018. Measurements included size-resolved PM fractions (PM_2.5_, PM_10–2.5_, and > PM_10_), short-lived α activity (SLA) and long-lived α activity (LLA) associated with PM_2.5_, elemental composition, black carbon (BC), and a biomass-burning/cooking surrogate (DC).

HEPA operation substantially reduced indoor particle concentrations, with mean decreases of 3.7, 1.0, and 0.5 μg/m^3^ for PM_2.5_, PM_10–2.5_, and >PM_10_, corresponding to removal efficiencies of approximately 60, 37, and 32%, respectively. LLA decreased by 0.68 mBq/m^3^, corresponding to a 63% removal efficiency (**p** < 0.001). SLA also decreased substantially, by 31.4 Bq/m^3^, or approximately 70%; however, this reduction was not statistically significant, likely because of the limited number of valid SLA measurements. Major and trace PM_2.5_ constituents – including sulfur, crustal elements, BC, and DC-also decreased significantly during HEPA operation.

By jointly assessing PM mass, chemical composition, and particle-bound radioactivity across seasons (summer versus winter) and residential locations (family rooms versus basements), this study extends previous chamber-based and limited field investigations of air-cleaner performance. Overall, the findings indicate that portable HEPA cleaners can reduce indoor PM, chemically diverse PM_2.5_ constituents, and particle-bound radon decay products in occupied homes. HEPA filtration may therefore provide a practical supplemental strategy for reducing combined exposures to PM and particle-bound radioactivity. However, it does not remove gaseous radon and should not replace radon source-control or remediation measures.

## 1. Introduction

Indoor environments contain many particle sources, including cooking, space heating, and cleaning, along with infiltration of outdoor particles through the building envelope and ventilation systems (Tang et al., 2018; Vardoulakis et al., 2020; U.S. EPA, 2025). Recent residential construction and weatherization have emphasized airtight envelopes to improve energy efficiency. While reduced leakage can lower energy use, it may also increase indoor particulate matter (PM) concentrations and related health risks if not accompanied by adequate ventilation and air cleaning.

Indoor PM spans a broad size range and is commonly categorized as fine (PM_2.5_, <2.5 μm), coarse (PM_10–2.5_, 2.5–10 μm), and >PM_10_ (>10 μm). Fine particles are of particular concern because they penetrate deeply into the respiratory tract and reach the gas-exchange regions of the lungs. Beyond their intrinsic toxicity, indoor particles can carry other hazardous agents, including radioactive species, thereby amplifying health impacts. Numerous epidemiological and toxicological studies link PM_2.5_ exposure to respiratory and cardiovascular disease, reduced lung function, increased hospital and emergency visits, and mortality (Chen et al., 2021; Nuwongcharoen et al., 2023; Li et al., 2024).

Radon, a radioactive noble gas with a 3.8-day half-life, is generated by uranium decay in rocks and soils and can accumulate indoors, especially in basements and ground-contact rooms. Although radon itself is chemically inert and largely exhaled, its decay products are metal ions that readily attach to airborne particles, forming particle-bound radioactivity (PR). This progeny, including the short-lived α emitters ^218^Po and ^214^Po and the long-lived α emitter ^210^Po, emit α radiation that can damage airway epithelium and increase lung cancer risk (U.S. EPA, 2003; WHO, 2009; NCI, 2025). Immediately after formation, decay products exist as unattached ultrafine clusters (0.5–5 nm) for less than about 1 s, then rapidly attach to larger particles within 1–100 s (Offermann et al., 1984; Porstendörfer, 1994). Once attached, the host particles are radioactive; upon inhalation, emitted α particles can interact with lung tissue and other organs, causing DNA damage and raising lung cancer risk (NRC, 1988; WHO, 2021). In many countries, indoor exposure to radon and its decay products accounts for roughly half of all nonmedical radiation exposure (UNSCEAR, 2000; WHO, 2021). Emerging evidence also links radon and its progeny to adverse fetal, perinatal, and maternal outcomes, reduced pulmonary function and COPD-related morbidity and mortality in adults, and asthma morbidity in children (Wang et al., 2023; Banzon et al., 2025; Nyhan et al., 2019; Papatheodorou et al., 2021).

Air filtration is one of the most accessible strategies for controlling residential particulate pollution. Portable HEPA cleaners are relatively inexpensive, easy to deploy, and have been shown to substantially reduce indoor PM_2.5_ and related particle metrics in occupied homes (Allen et al., 2017; Waring et al., 2022). However, most studies focus on changes in particle mass, number, or short-term health indicators, and few have examined how HEPA filtration affects indoor particle radioactivity or the behavior of radon decay products. In particular, the extent to which HEPA cleaners alter the distribution of particle-bound radioactivity across size fractions and chemical components in real homes remains poorly understood.

In this study, one of the few real-world evaluations in occupied residence, we assessed the effectiveness of a portable HEPA cleaner, with emphasis on its impact on (i) size-resolved indoor particle concentrations, (ii) particle chemical composition, and (iii) PR associated with radon decay products. By simultaneously characterizing changes in particulate pollution and PR, we aim to clarify the potential of residential HEPA filtration to reduce indoor exposures associated health risks from PM and radon decay products.

## 2. Methods

### 2.1. Sampling scheme

We collected 86 indoor samples in a convenience sample of 19 homes in the metropolitan Boston area between June 2017 and March 2018. Each home was sampled for 10 days per season in the family room, with 5 days without and 5 days with a HEPA cleaner operating, in either the warm or cold season. Five single–family homes were randomly selected for more intensive sampling, with concurrent measurements in the family room and the basement.

Accordingly, we implemented two sampling configurations (Fig. 1): (i) a regular measurement and (ii) an intensive measurement. For the regular measurements, a cascade indoor sampler, radon charcoal canisters (Radon testing corporation of America, NY), and a CO_2_ sensor were placed in the family room, where residents spent most of their time. For the intensive measurements, we obtained additional measurements of short–lived α–activity (SLA) in both the family room and the basement, where higher levels of radiation exposure were expected. All basements were similarly unfurnished. The CO_2_ sensor (Model AZ0003, Global Sensors, Ormond Beach, FL) with a non–dispersive infrared diffusion sensor was placed next to the indoor samplers to provide real–time indoor measurements of CO_2_, temperature, and relative humidity.

In total, samples were collected in 19 homes (including 5 with intensive measurements) in summer and 14 homes (including 4 with intensive measurements) in winter. Some residents did not participate in follow–up winter sampling. Field staff visited homes three times per campaign: (i) to set up sampling equipment on day 1, (ii) to change samples and install the air cleaner on day 5, and (iii) to remove samplers on day 10. Because of logistical constraints and occasional instrument malfunction, fewer valid SLA samples were collected than other measurements.

### 2.2. Short– and long–lived α–activities

Short–lived particle α–activity (SLA) from radon decay products (^218^Po and ^214^Po) was measured using an electret radon decay products integrating sampling unit (E–RPISU; Rad Elec Inc., Frederick, MD). The unit collects radon decay products on a 25–mm filter at a low flow rate of 0.8 L/min and registers α–radiation from the deposited decay products during sample collection (Kotrappa et al., 2003). This method has been widely used to monitor SLA from α–radiation emitted by radon decay products captured on filters (US DOE, 1994; Dua et al., 1999).

Long–lived particle α–activity (LLA) from radon decay products (^210^Po) was measured on the PM_2.5_ filters. Filters were stored for more than one year to allow ingrowth of ^210^Po from ^210^Pb and then analyzed using a low–background proportional counter (Model LB4200, Canberra Industries, Inc., Meriden, CT) with a P10 carrier gas (10% methane in argon). Because of the low radioactivity of the filter samples, we used a 600–minute counting time to ensure sufficient counts for reliable quantification. Using a 0.0518 μCi NIST–traceable ^210^Po source on a 5.7–cm planchet, the gross 4π α-counting efficiency was 40 ± 1%. The detection limit was 0.015 mBq/m^3^ for a sample volume of 36 m^3^. Detailed descriptions of this analysis are provided elsewhere (Kang et al., 2020).

### 2.3. PM mass and PM_2.5_ components

Indoor PM samples were collected using two–stage cascade impactors that capture particles in three size ranges at a flow rate of 5 L/min. The two upstream impactor stages are equipped with slit–shaped acceleration nozzles and are followed by a backup Teflon filter at the third stage. Polyurethane foam (PUF) substrates were used as impaction surfaces on the first two stages to collect particles > 10 μm (>PM_10_) and 2.5–10 μm (PM_10–2.5_) (Case et al., 2008; Lee et al., 2006). The third stage used a 37–mm Teflon filter supported by a drain disk and a stainless–steel screen to collect PM_2.5_.

Teflon filters were weighed before and after sampling using an electronic microbalance (Model MT–5, Mettler Toledo, Columbus, OH) under controlled temperature and relative humidity to obtain gravimetric mass concentrations. Filters were subsequently analyzed for sulfur and other elements using an energy–dispersive X–ray fluorescence (EDXRF) spectrometer (Model Epsilon 5, PANalytical, The Netherlands) (Kang et al., 2014). The same filters were also used for LLA analysis as described above.

To characterize carbonaceous fractions, we used an optical transmissometer (OT21, Magee Scientific), which allows retrospective filter–based estimation of black carbon (BC) and brown carbon (BrC). This approach is analogous to multichannel wavelength aethalometers used for real–time monitoring. Enhanced absorption at ultraviolet wavelengths (e.g., 370 nm) reflects aromatic organic compounds from sources such as wood smoke, biomass burning, and tobacco smoke (BrC), whereas absorption at near–infrared wavelengths (e.g., 880 nm) is dominated by BC from traffic emissions. Because BC and BrC are typically correlated, we used the difference (Delta Carbon, DC = BrC – BC) as a surrogate for biomass burning.

### 2.4. HEPA cleaner and effectiveness

We used portable HEPA cleaners. Each unit includes four filtration stages: a pre–screen to remove hair and dust fibers, a pre–filter to capture large particles, a metal–embedded activated carbon bed to remove odors and volatile organic compounds, and a HEPA filter to remove fine particles. After the summer campaign and before new home setting, activated carbon and HEPA filters were replaced, and the pre–screen mesh and pre–filter were cleaned using a vacuum cleaner.

The air cleaner was placed opposite the indoor samplers in the family room, as far apart as practical. According to the manufacturer, a single unit covers up to 145 m^2^ of living space, with a clean air delivery rate (CADR) of 9.9 m^3^/min. For a typical room volume, this corresponds to several clean–air changes per hour. Because the same model was operated at the same (or similar) airflow rate in all homes, we considered the particle removal efficiency of the units to be comparable across houses. The removal efficiency (ηij, %) for a specific home (i) during a given season (j) was calculated as:

$$\eta ij=\frac{C_{off,ij}\;-\;C_{on,ij}}{C_{off,ij}}\times 100\%$$

where $C_{off,ij}$ is the concentration before the intervention (HEPA off) and $C_{on,ij}$ is the concentration during the intervention (HEPA on) for a specific home (i) during a given season (j).

## 3. Results

Nineteen homes (9 single-families, 10 multifamily residences) participated in the Boston air cleaner intervention study. The homes were constructed between 1900 and 2016, and their mean living area was approximately 139 m^2^, making them broadly representative of housing in the northeastern United States. Indoor measurements collected before HEPA deployment served as the control for each home and season. Table 1 summarizes the mean control concentrations and changes observed during the intervention. Because HEPA filtration is not expected to remove gas-phase radon and our analysis focused on particulate-phase constituents, gas-phase radon was not included in subsequent analyses; however, these data are provided in the tables.

### 3.1. Particle size distribution

Baseline indoor averages of PM_2.5_, PM_10–2.5_, and >PM_10_ were 5.9, 2.9, and 2.4 μg/m^3^, respectively (Table 1). During HEPA operation, the corresponding averages decreased to 2.2, 1.9, and 1.9 μg/m^3^. The reductions of 3.7 and 1.0 μg/m^3^ for PM_2.5_ and PM_10–2.5_ were statistically significant (p < 0.01), whereas the reduction of 0.5 μg/m^3^ for >PM_10_ was not statistically significant (Fig. 2). All particle size fractions decreased during HEPA operation, with the largest absolute and relative reductions observed for PM_2.5_, followed by PM_10–2.5_ and >PM_10_. This pattern indicates that apparent removal efficiency decreases with increasing particle size, presumably because coarse particles, which are mainly generated indoors, are not well mixed.

### 3.2. Particle radioactivity and elemental composition

Average SLA and LLA during non-intervention periods were 43.2 Bq/m^3^ and 1.07 mBq/m^3^, respectively. As expected, SLA exceeded LLA because short-lived progeny decay faster, yielding higher specific activity. With HEPA operation, SLA and LLA averages fell to 11.8 Bq/m^3^ and 0.39 mBq/m^3^, corresponding to reductions of 31.4 Bq/m^3^ and 0.68 mBq/m^3^ (Table 1). The LLA reduction was statistically significant (p < 0.001), whereas the large SLA reduction was not, due to the limited number of SLA samples. Although SLA and LLA represent different particle size domains (non–size-selective PM vs. PM_2.5_), radioactivity is expected to be dominated by fine particles because of their larger surface area (Liu et al., 2018). The estimated removal efficiency for LLA (63%) and the point estimate for SLA (70%) indicate that particle-bound decay products are removed at least as efficiently as nonradioactive aerosol, though the SLA estimate is more uncertain.

BC (traffic surrogate) and DC (biomass-burning surrogate) averaged 0.32 and 0.12 μg/m^3^ during control periods and decreased to 0.14 and 0.08 μg/m^3^ with HEPA use, yielding statistically significant reductions of 0.18 μg/m^3^ (BC, p < 0.001) and 0.05 μg/m^3^ (DC, p < 0.01). Among PM_2.5_ elements, sulfur was most abundant, averaging 194 ng/m^3^ during control periods and decreasing to 69.7 ng/m^3^ with HEPA, a reduction of 124 ng/m^3^. Na, K, and Si (sea salts and crustal elements) averaged 67.6, 49.1, and 31.8 ng/m^3^, with reductions of 44.6, 36.7, and 17.8 ng/m^3^. Trace elements ranged from 0.9 ng/m^3^ (Br, infiltrated marine aerosol, traffic- or combustion-related emissions, and indoor brominated materials) to 29.5 ng/m^3^ (Cl, infiltrated outdoor salt aerosols and indoor activities such as cooking and chlorine-based cleaning) before the intervention and showed significant decreases (e.g., 17.3 ng/m^3^ for Cl, 0.4 ng/m^3^ for Br); most elemental reductions were significant at p < 0.001 or p < 0.01, except Pb.

Radon concentrations varied somewhat, possibly because of the cleaner’s charcoal filter and/or day-to-day variation. Because portable HEPA cleaners are not expected to remove gas-phase radon from indoor air, we excluded these results from further discussion to avoid misinterpretation. CO_2_ concentrations, temperature, and relative humidity were similar before and after HEPA deployment (Tables S1 and S2), indicating that the intervention primarily affected particles rather than ventilation or indoor thermal conditions.

### 3.3. Effects of seasons and house locations

Indoor measurements were conducted in summer (June–September 2017) and winter (December 2017–March 2018). Tables S1 and S2 also summarize seasonal concentrations and those by location (family rooms vs. basements). In summer, average PM_2.5_, PM_10–2.5_, >PM_10_, and most other species were higher, likely due to increased air exchange from more frequent window and door opening. In contrast, DC, Cl, Zn, and Pb were higher in winter; elevated Cl is consistent with road salt, and higher DC suggests increased biomass burning (e.g., wood stoves).

Most species showed larger absolute HEPA effects in summer. For PM_2.5_, PM_10–2.5_, and >PM_10_, HEPA operation reduced concentrations by 4.5, 1.2, and 0.8 μg/m^3^ in summer versus 2.6, 0.5, and 0.1 μg/m^3^ in winter. For SLA and LLA, reductions were 36.3 Bq/m^3^ and 0.80 mBq/m^3^ in summer and 20.6 Bq/m^3^ and 0.51 mBq/m^3^ in winter. LLA reductions in both seasons were statistically significant (p < 0.001), whereas SLA reductions were not, given the small sample size. BC and most elements showed similar seasonal patterns, while DC and Al had greater absolute reductions in winter, reflecting higher winter baselines. Paired t-tests, however, did not identify statistically significant seasonal differences in reductions.

Concentrations also varied by location. Except for SLA (substantially higher in basements: 60.5 Bq/m^3^) and LLA, most measurements were roughly twice as high in family rooms as in basements, consistent with greater activity and air exchange in primary living spaces. Because all elements were analyzed on PM_2.5_ filters, component-to-PM_2.5_ ratios generally followed PM_2.5_, except for a few low-concentration species (DC, Br, Pb). Reduction patterns by location broadly resembled those by size fraction. In basements, HEPA airflow may have resuspended coarse particles, contributing to relatively small reductions (or slight increases) in >PM_10_ compared with PM_10–2.5_. LLA reductions were larger in basements than in family rooms, reflecting higher baseline radioactivity; limited valid SLA data in family rooms precluded location-specific SLA estimates, though larger absolute SLA reductions are expected in basements.

For PM_2.5_, the HEPA intervention reduced concentrations by 4.2 μg/m^3^ in family rooms and 2.0 μg/m^3^ in basements, with many elements showing larger reductions in family rooms, consistent with PM_2.5_. Although point estimates varied by season and location for several species, overlapping confidence intervals suggest these differences should be interpreted as indicative rather than definitive evidence of differential HEPA effects.

## 4. Discussion

We evaluated a portable HEPA cleaner’s impact on indoor particle mass, size fractions, chemical components, and associated PR in occupied homes in the Boston area, quantifying reductions from the difference between control and intervention measurements (Fig. 2).

The HEPA cleaner substantially reduced indoor particulate matter, with the largest effect on fine particles (PM_2.5_). Average PM_2.5_ fell from 5.9 to 2.2 μg/m^3^ (~ 60% removal), while reductions for PM_10–2.5_ and > PM_10_ were more modest (37.1% and 31.9%). Our findings agree with prior residential, chamber, and crossover studies reporting size-dependent removal efficiencies of 30–70% and PM_2.5_ reductions of 30–40% from portable HEPA units in homes and classrooms (Brugge et al., 2025; Jhun et al., 2017; Simon et al., 2025). Smaller coarse-particle reductions may also reflect poorer mixing and diverse sources (e.g., skin flakes, fibers, resuspended dust, and outdoor dust/soil).

The intervention also strongly reduced PR. LLA from long-lived decay products (^210^Po) on PM_2.5_ decreased with an estimated 62.7% removal efficiency and high statistical significance. SLA from short-lived decay products (^218^Po, ^214^Po) showed a large average reduction (~70% removal) but with imprecise, non-significant estimates due to the small sample size. Overall, the pattern suggests that particle-bound radon decay products are at least as efficiently removed as nonradioactive aerosols. Fresh decay products form mobile, charged nanoclusters that rapidly attach to aerosol and plate out on surfaces (Porstendörfer, 1994; Kotrappa et al., 2003; NRC, 1988); by lowering airborne particle number and surface area, HEPA filtration removes attached decay products and facilitates deposition of unattached ions and clusters. Previous work (Kang et al., 2020) showed SLA is closely linked to indoor radon, while LLA tracks sulfur (a marker of infiltrated outdoor particles), reinforcing that efficient filtration can substantially reduce the component of indoor radiation dose mediated by particle-bound decay products. These reductions are relevant for lung cancer risk (National Research Council (NRC), 1988; UNSCEAR, 2000; WHO, 2009; U.S. EPA, 2003) and emerging non-cancer outcomes in vulnerable groups. However, HEPA filtration does not reduce radon gas itself, only the particle-bound portion of the dose.

Chemical composition measurements further support the effectiveness of HEPA filtration. Sulfur decreased by 124.3 ng/m^3^, corresponding to a removal efficiency of 66.9%, similar to that of PM_2.5_. Because sulfur in indoor fine particles is often associated with infiltrated outdoor aerosol, this reduction is consistent with effective removal of outdoor-derived sulfur-containing particles. Significant decreases in Na, K, Si, Ca, Fe, and several trace elements further indicate that the cleaner removed particles with diverse chemical characteristics. BC and DC, which serve as indicators of traffic-related combustion and biomass-burning/cooking influences, decreased by approximately 56.1% and 48.8%, respectively. The observed reductions are broadly consistent with the major indoor PM_2.5_ source categories previously identified in Boston – indoor mixed dust, sea/road salts, biomass burning, and outdoor air pollution (Matthaios et al., 2021). Although source apportionment was beyond the scope of this study, these results suggest that HEPA filtration can reduce PM_2.5_ associated with multiple indoor and outdoor sources. Together, the findings align with prior work showing that indoor fine particle composition in residences and schools often reflects substantial outdoor contributions, even in relatively airtight buildings (Derbez et al., 2018; Jhun et al., 2017; Langer et al., 2016; Simon et al., 2025; Tang et al., 2018).

Seasonal analyses (Fig. 3) highlight the role of infiltration and sources. Most particle and elemental concentrations, as well as LLA, were higher in summer than in winter, consistent with greater window opening and higher air exchange rates (ASHRAE, 1981; Derbez et al., 2018). Accordingly, absolute HEPA-related reductions in PM_2.5_, SLA, LLA, BC, and many elements were generally larger in summer, indicating that the cleaner removes more mass when outdoor-derived pollutant loads and indoor–outdoor exchange are higher. These patterns agree with prior radon and radon decay studies and HEPA intervention work across different outdoor pollution regimes (Dudney et al., 1990; Kang et al., 2020; Simon et al., 2025).

Location contrasts (Fig. 4) underscore microenvironmental differences. Family rooms had roughly twice the concentrations of most PM_2.5_ components as basements, reflecting more activity, higher air exchange, and greater outdoor influence (Wallace et al., 1991; Robinson and Thomas, 1991; Langer et al., 2016). In contrast, radon and its decay products (SLA) were much higher in basements, consistent with proximity to soil gas entry. Nevertheless, the HEPA cleaner significantly reduced LLA in both locations, with larger estimated PM_2.5_ and elemental reductions in family rooms, likely due to higher baselines and more resuspension.

Our results are consistent with a growing intervention literature showing that HEPA filtration not only reduces indoor PM_2.5_ but can also yield short-term health benefits. Recent randomized trials in homes and schools have reported improved blood pressure in adults and better lung function and reduced airway inflammation in children following HEPA interventions (Brugge et al., 2025; Li and Kan, 2024). Although we did not measure health outcomes, this evidence, combined with our findings on PM_2.5_, composition, and particle radioactivity, suggests that portable HEPA cleaners are a practical near-term strategy to reduce inhalation exposures in existing buildings, particularly where radon potential, traffic or biomass-related pollution, and airtight, energy-efficient housing are concerns.

The primary novelty of this study is its integrated assessment of the effects of portable HEPA filtration on indoor particulate matter, particle chemical composition, and particle-bound radioactivity across seasons (summer versus winter) and residential locations (family rooms versus basements) in occupied homes. Unlike most residential air-cleaner studies, which focus primarily on PM_2.5_ mass or short-term health indicators, we measured both long-lived and short-lived α-activity associated with radon decay products, together with size-fractionated PM and elemental and carbonaceous constituents. Our results demonstrate that portable filtration substantially reduces long-lived particle-bound α-activity, with removal efficiencies comparable to those observed for PM_2.5_, while leaving gaseous radon largely unaffected. These findings extend previously controlled or limited investigations by providing field-based evidence that particle filtration can reduce a potentially important component of combined indoor PM and radon-progeny exposure.

## 5. Conclusions

Indoor radon and its decay products (SLA, LLA) are important health concerns because they increase lung cancer risk, and coexisting fine particles can amplify this risk by carrying radon progeny. In our study, indoor particle radioactivity varied by home and season but was consistent with prior work, supporting our methods.

Portable HEPA cleaners substantially reduced indoor PM_2.5_, key components (sulfur and traffic/biomass markers), and both short- and long-lived α activity from radon decay products. Removal efficiencies for particle radioactivity were comparable to or greater than those for PM_2.5_, indicating strong control of particle-bound radiation. Reductions in long-lived α activity were precise and statistically robust, while decreases in short-lived α activity, though often large, were more uncertain.

Overall, portable HEPA cleaners are a practical and effective way to lower exposures to fine particles and PR in homes, potentially mitigating related health risks, especially where radon or outdoor PM infiltration is high. However, because HEPA filtration does not remove radon gas, comprehensive mitigation still requires source control and building-level remediation in homes with elevated radon.

## Supplementary Material

Supplement

## Figures and Tables

**Fig. 1.**
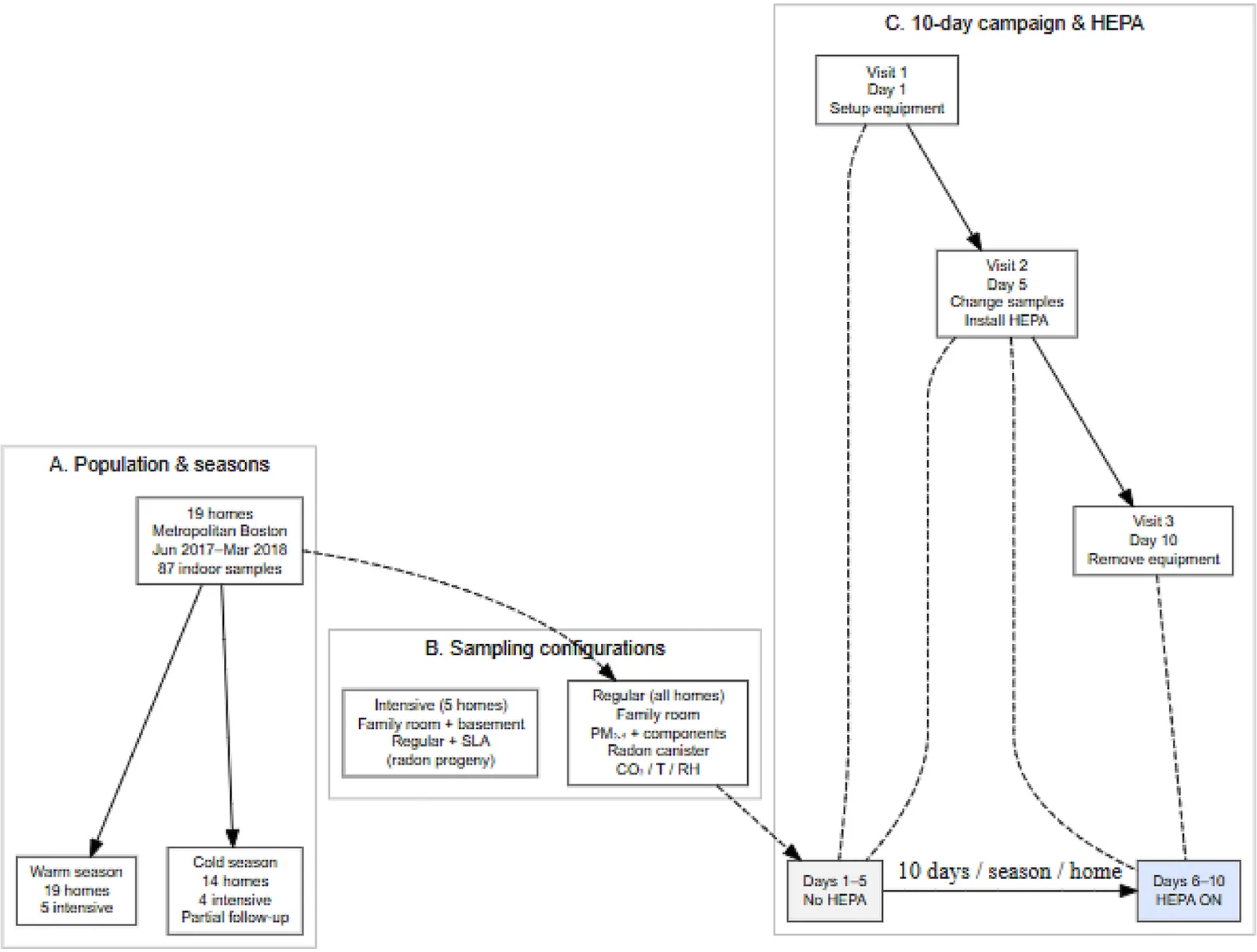
Study Design.

**Fig. 2.**
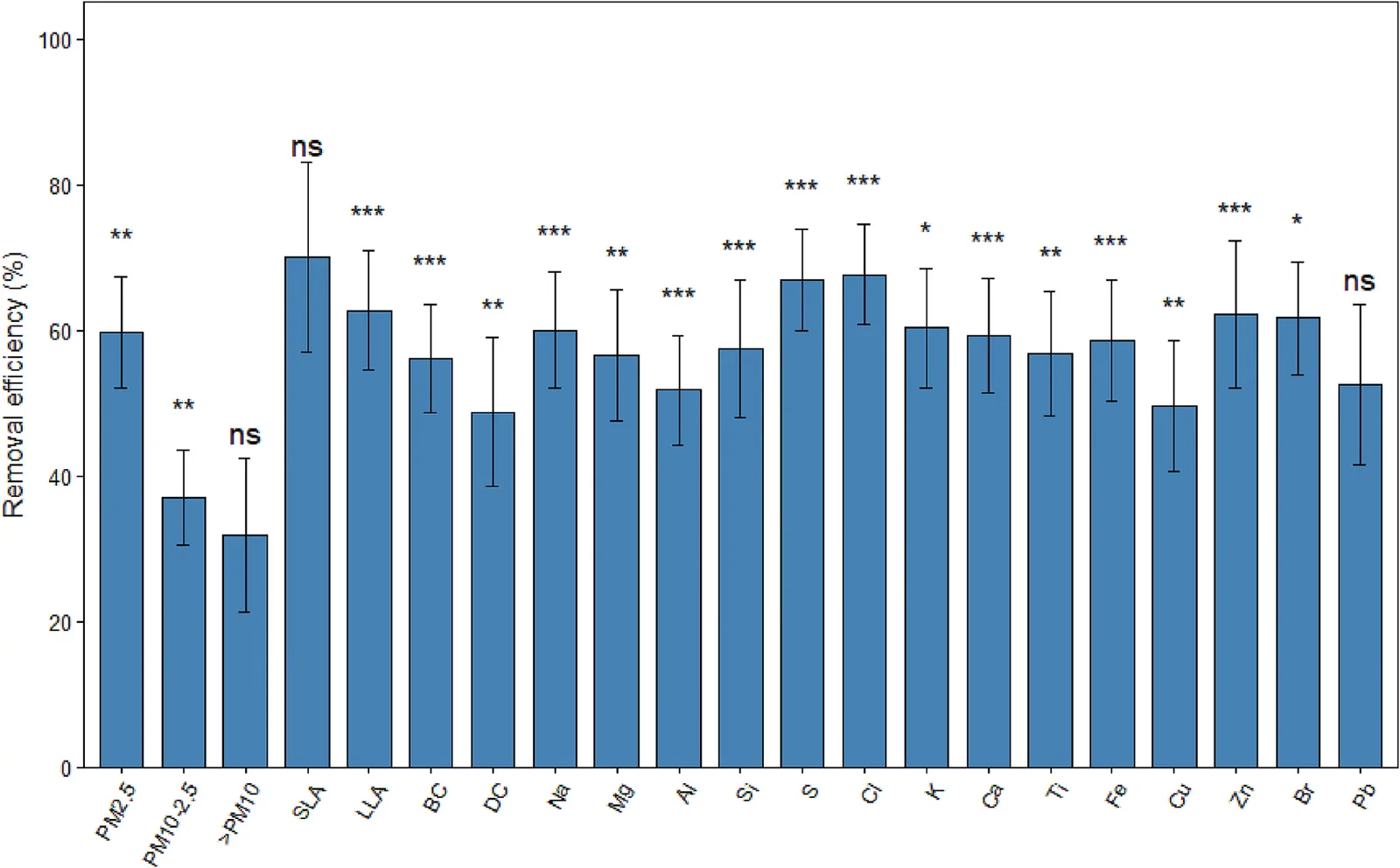
Intervention reduction by species — In this plot, bars indicate mean removal efficiency for each paired species, defined as (control – intervention)/control × 100%; whiskers indicate ± 2 standard error; p-value from paired ON vs. OFF t-tests.

**Fig. 3.**
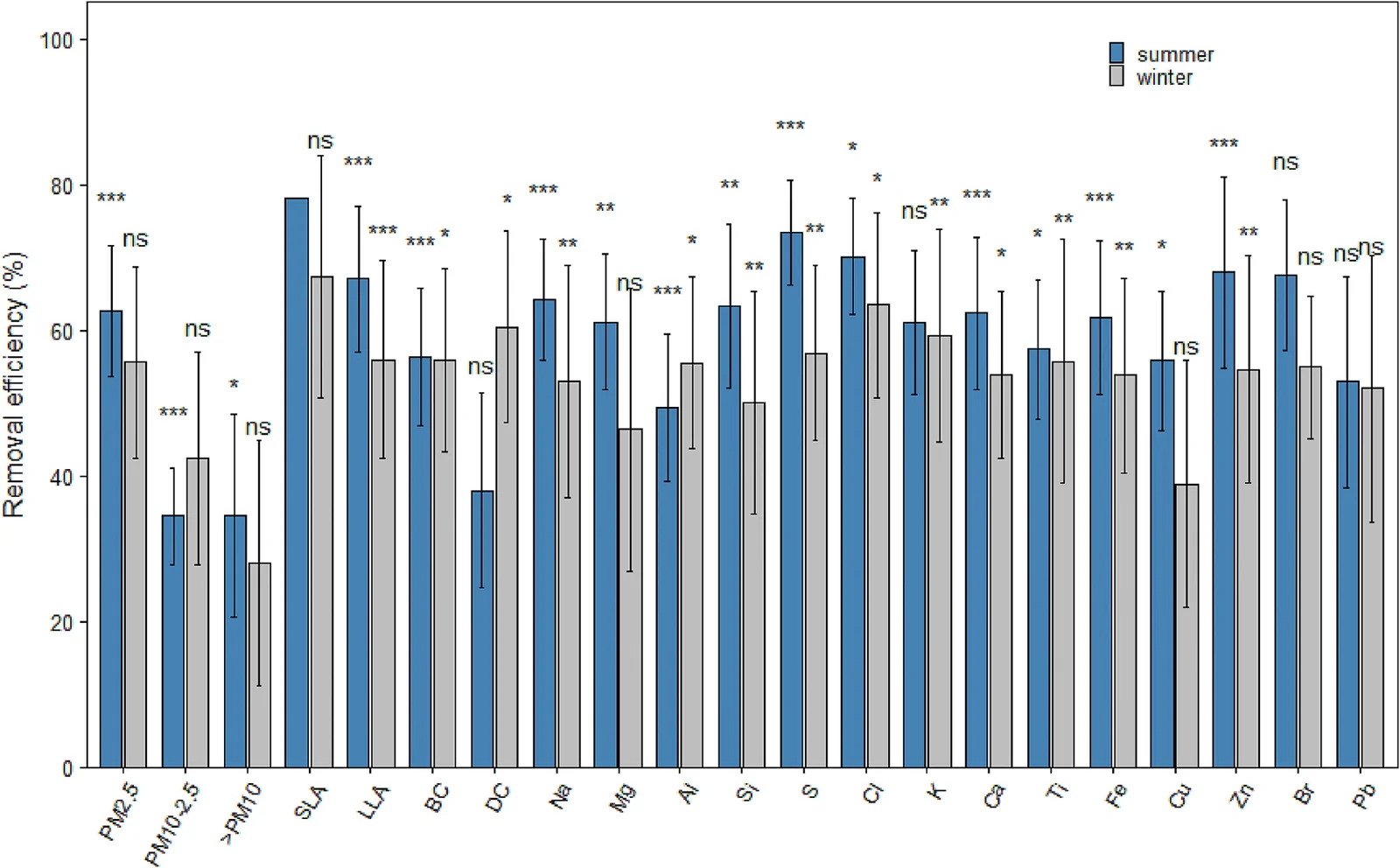
Intervention reduction by seasons — In this plot, bars indicate mean removal efficiency for each paired species, defined as (control – intervention)/control × 100%; whiskers indicate ± 2 standard error; p-value from paired ON vs. OFF t-tests.

**Fig. 4.**
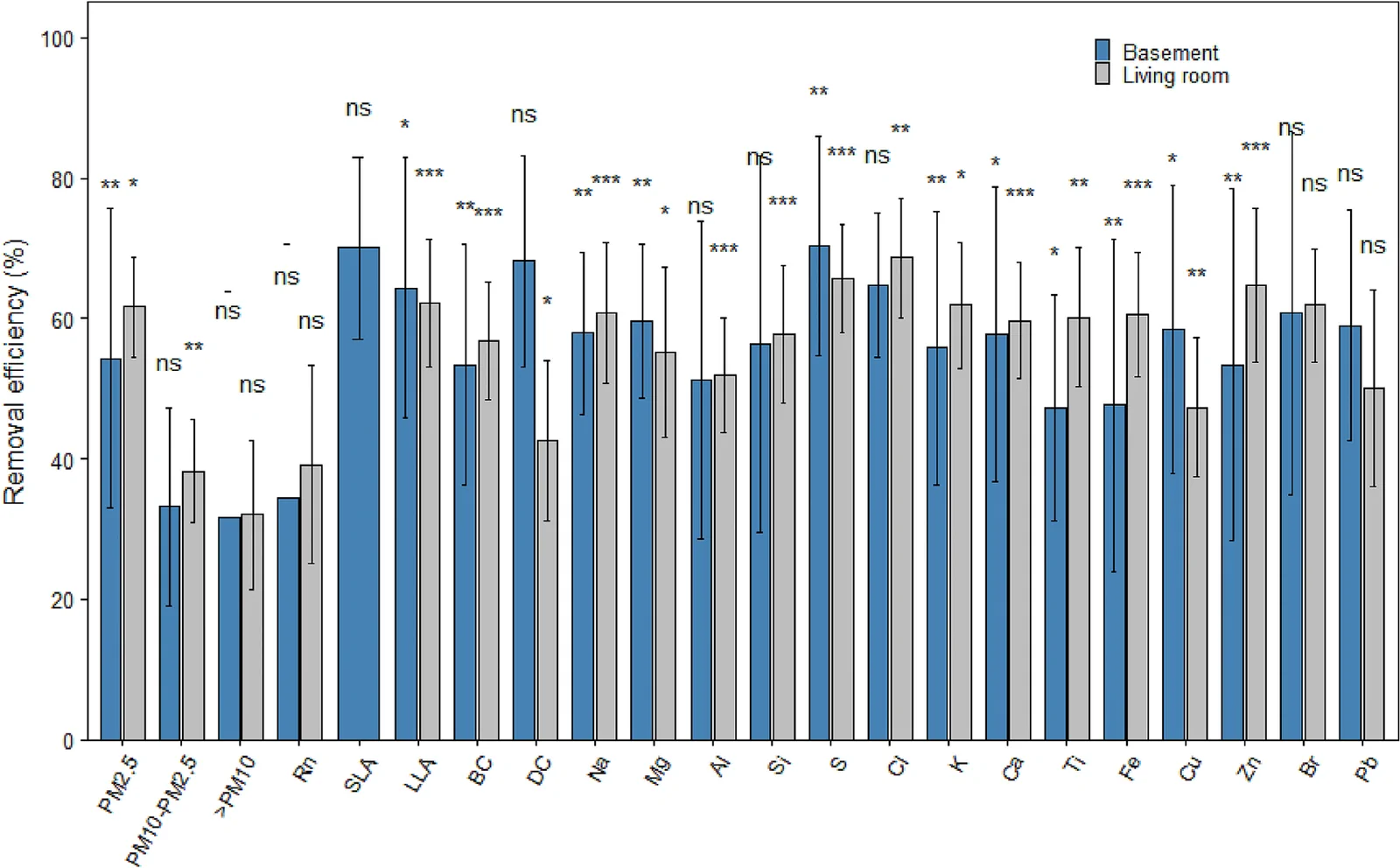
Intervention reduction by house locations — In this plot, bars indicate mean removal efficiency for each paired species, defined as (control – intervention)/control × 100%; whiskers indicate ± 2 standard error; p-value from paired ON vs. OFF t-tests.

**Table 1:** Overall measurements of control and intervention.

Species	Control (AC=OFF)						Intervention (AC=ON)				
			
	Mean	SD^1^	Min	Max	N^2^		Mean	SD	Min	Max	N

PM_2.5_ (μg/m^3^)	5.9	3.5	1.0	15.1	44		2.2	1.4	0.2	5.5	42
PM_10–2.5_ (μg/m^3^)	2.9	1.7	0.3	6.4	44		1.9	1.0	0.4	4.8	42
>PM_10_ (μg/m^3^)	2.4	1.3	0.5	5.2	44		1.9	0.9	0.5	4.0	42
Rn (Bq/m^3^)	50.4	91.7	3.7	537	43		44.3	85.2	3.7	488	41
SLA (Bq/m^3^)	43.2	39.1	5.3	131	12		11.8	5.8	4.4	17.3	4
LLA (mBq/m^3^)	1.07	0.52	0.09	2.72	43		0.39	0.31	0.0	1.33	41
BC (μg/m^3^)	0.32	0.17	0.06	0.93	44		0.14	0.08	0.00	0.35	42
DC (μg/m^3^)	0.12	0.08	0.04	0.50	44		0.08	0.04	0.01	0.21	42
Na (ng/m^3^)	67.6	63.6	0.0	274	44		23.0	22.5	0.0	105	42
Mg (ng/m^3^)	14.1	13.4	0.0	82.4	44		6.7	4.1	0.0	16.1	42
Al (ng/m^3^)	18.3	12.1	0.7	64.7	44		8.8	5.1	0.0	21.4	42
Si (ng/m^3^)	31.8	28.3	3.4	166	44		14.0	11.6	0.4	44.5	42
S (ng/m^3^)	194	110	17.6	436	44		69.7	58.9	0.0	236	42
Cl (ng/m^3^)	29.5	42.2	0.2	157	44		12.2	21.1	0.1	119	42
K (ng/m^3^)	49.1	97.6	4.4	662	44		12.4	8.5	0.1	31.6	42
Ca (ng/m^3^)	23.7	20.4	2.8	95.5	44		9.8	7.5	0.0	32.8	42
Ti (ng/m^3^)	2.4	2.0	0.1	15.2	44		1.0	0.7	0.1	3.2	42
Fe (ng/m^3^)	20.9	17.1	0.7	82.2	44		7.7	7.7	0.0	30.9	42
Cu (ng/m^3^)	3.7	3.9	0.5	25.6	44		2.0	2.2	0.2	14.4	42
Zn (ng/m^3^)	4.2	3.1	0.0	19.0	44		1.4	1.2	0.0	4.7	42
Br (ng/m^3^)	0.9	0.7	0.0	2.3	44		0.5	0.5	0.0	2.3	42
Pb (ng/m^3^)	2.8	3.1	0.0	20.0	44		2.1	1.2	0.0	4.4	42
CO_2_ (ppm)	569	135	399	1135	43		571	141	423	1177	38
Temp (°C)	22.1	3.3	12.7	27.1	44		22.3	3.5	13.1	28.1	39
RH (%)	48	16	14	87	44		47	15	19	83	39

1 Standard deviation

2 Number of measurements

## Data Availability

Data will be made available on request.

## Appendix A. Supplementary data

Supplementary data to this article can be found online at https://doi.org/10.1016/j.envint.2026.110528.
