# Supplementary material for: Portable HEPA air filtration effects on particulate matter, radioactivity, and chemical constituents in occupied homes
Source: Environ Int. Author manuscript; Available in PMC 2026 Oct 17. (PMC13670566; doi:10.1016/j.envint.2026.110528)
Supplement: Supplementary file 1 [file NIHMS2212915-supplement-Supplement.docx]

**SUPPORTING INFORMATION**

**Table A1. Control and intervention measurements by seasons.**

| Species | Summer | | | Winter | | |
| --- | --- | --- | --- | --- | --- | --- |
|  | Control | Intervention | N^1^ | Control | Intervention | N |
| PM_2.5_ (µg/m^3^) | 7.1±3.6^2^ | 2.6±1.7 | 51 | 4.2±2.5 | 1.6±0.7 | 35 |
| PM_10-2.5_ (µg/m^3^) | 3.3±1.7 | 2.1±1.1 | 51 | 2.2±1.4 | 1.7±0.8 | 35 |
| >PM_10_ (µg/m^3^) | 2.7±1.4 | 1.9±0.9 | 50 | 1.9±1.1 | 1.8±1.0 | 35 |
| Rn (Bq/m^3^) | 33.2±59.4 | 30.7±53.8 | 50 | 76.6±124 | 63.6±115 | 34 |
| SLA (Bq/m^3^) | 51.7±47.9 | 15.4^3^ | 8 | 31.3±21.3 | 10.7±6.4 | 8 |
| LLA (mBq/m^3^) | 1.20±0.55 | 0.40±0.34 | 49 | 0.88±0.41 | 0.37±0.27 | 35 |
| BC (µg/m^3^) | 0.34±0.15 | 0.14±0.08 | 51 | 0.30±0.18 | 0.13±0.08 | 35 |
| DC (µg/m^3^) | 0.09±0.02 | 0.07±0.03 | 51 | 0.17±0.10 | 0.08±0.05 | 35 |
| Na (ng/m^3^) | 68.7±65.3 | 19.8±21.0 | 51 | 65.8±62.9 | 27.8±24.2 | 35 |
| Mg (ng/m^3^) | 17.4±15.8 | 6.9±4.3 | 51 | 9.4±6.6 | 6.4±3.9 | 35 |
| Al (ng/m^3^) | 22.0±13.7 | 9.2±5.2 | 51 | 13.1±6.7 | 8.1±5.2 | 35 |
| Si (ng/m^3^) | 41.2±32.8 | 16.3±12.8 | 51 | 18.3±10.8 | 10.6±8.8 | 35 |
| S (ng/m^3^) | 231±120 | 77.9±69.1 | 51 | 140±67.2 | 57.7±38.5 | 35 |
| Cl (ng/m^3^) | 21.0±41.3 | 5.6±12.7 | 51 | 41.7±41.6 | 21.8±27.1 | 35 |
| K (ng/m^3^) | 56.3±125 | 12.5±9.9 | 51 | 38.6±30.8 | 12.1±6.1 | 35 |
| Ca (ng/m^3^) | 26.1±21.1 | 9.3±6.9 | 51 | 20.2±19.4 | 10.5±8.5 | 35 |
| Ti (ng/m^3^) | 2.9±2.8 | 1.1±0.8 | 51 | 1.7±0.9 | 0.9±0.6 | 35 |
| Fe (ng/m^3^) | 25.5±19.5 | 8.7±8.9 | 51 | 14.3±10.1 | 6.3±5.6 | 35 |
| Cu (ng/m^3^) | 4.3±4.8 | 2.2±2.8 | 51 | 2.8±2.0 | 1.6±0.7 | 35 |
| Zn (ng/m^3^) | 3.2±1.5 | 1.2±1.2 | 51 | 5.7±4.2 | 1.8±1.2 | 35 |
| Br (ng/m^3^) | 1.0±0.7 | 0.6±0.6 | 51 | 0.8±0.7 | 0.5±0.4 | 35 |
| Pb (ng/m^3^) | 2.6±1.6 | 2.3±1.2 | 51 | 3.1±4.5 | 1.8±1.2 | 35 |
| CO_2_ (ppm) | 558±112 | 592±169 | 49 | 587±167 | 539±76 | 32 |
| Temp (ºC) | 23.7±2.2 | 24.2±2.2 | 49 | 19.9±3.3 | 19.5±3.1 | 34 |
| RH (%) | 58±8 | 57±8 | 49 | 32±12 | 33±10 | 34 |
| ^1^ Number of measurements  ^2^ Mean ± standard deviation  ^3^ A single measurement | | | | | | |

**Table A2. Control and intervention measurements by house locations.**

| Species | Family room | | | Basement | | |
| --- | --- | --- | --- | --- | --- | --- |
|  | Control | Intervention | N^1^ | Control | Intervention | N |
| PM_2.5_ (µg/m^3^) | 6.7±3.5^2^ | 2.5±1.5 | 65 | 3.3±1.9 | 1.3±0.8 | 21 |
| PM_10-2.5_ (µg/m^3^) | 3.4±1.6 | 2.3±1.0 | 65 | 1.2±0.4 | 0.9±0.4 | 21 |
| >PM_10_ (µg/m^3^) | 2.7±1.1 | 2.2±0.8 | 64 | 1.4±1.4 | 1.0±0.4 | 21 |
| Rn (Bq/m^3^) | 24.8±25.0 | 21.1±23.3 | 63 | 125±159 | 116±151 | 21 |
| SLA (Bq/m^3^) | 8.6±3.1 | NA^3^ | 4 | 60.5±37.0 | 11.8±5.8 | 12 |
| LLA (mBq/m^3^) | 1.07±0.53 | 0.41±0.32 | 64 | 1.04±0.49 | 0.32±0.30 | 20 |
| BC (µg/m^3^) | 0.34±0.18 | 0.14±0.08 | 65 | 0.24±0.07 | 0.13±0.09 | 21 |
| DC (µg/m^3^) | 0.12±0.08 | 0.08±0.03 | 65 | 0.12±0.07 | 0.07±0.05 | 21 |
| Na (ng/m^3^) | 76.3±69.0 | 26.2±24.2 | 65 | 37.8±24.9 | 13.9±13.6 | 21 |
| Mg (ng/m^3^) | 14.9±14.9 | 7.2±4.3 | 65 | 11.5±5.5 | 5.3±3.1 | 21 |
| Al (ng/m^3^) | 20.9±12.5 | 9.1±5.2 | 65 | 9.6±4.7 | 7.8±5.0 | 21 |
| Si (ng/m^3^) | 37.3±30.0 | 15.0±11.2 | 65 | 13.2±4.9 | 11.0±12.7 | 21 |
| S (ng/m^3^) | 206±118 | 78.1±63.5 | 65 | 151±66.7 | 46.2±36.1 | 21 |
| Cl (ng/m^3^) | 34.6±46.4 | 14.5±23.8 | 65 | 12.0±13.4 | 5.6±7.7 | 21 |
| K (ng/m^3^) | 56.2±110 | 13.4±8.6 | 65 | 24.8±14.3 | 9.5±7.6 | 21 |
| Ca (ng/m^3^) | 27.2±21.9 | 10.5±7.1 | 65 | 11.9±6.0 | 7.7±8.6 | 21 |
| Ti (ng/m^3^) | 2.7±2.5 | 1.1±0.8 | 65 | 1.2±0.6 | 0.7±0.5 | 21 |
| Fe (ng/m^3^) | 24.6±17.9 | 8.8±8.2 | 65 | 8.5±3.2 | 4.6±5.4 | 21 |
| Cu (ng/m^3^) | 4.3±4.3 | 1.9±1.0 | 65 | 1.8±0.7 | 2.3±4.1 | 21 |
| Zn (ng/m^3^) | 4.6±3.4 | 1.5±1.3 | 65 | 2.8±1.7 | 1.2±1.2 | 21 |
| Br (ng/m^3^) | 1.0±0.7 | 0.6±0.5 | 65 | 0.8±0.8 | 0.3±0.3 | 21 |
| Pb (ng/m^3^) | 2.9±3.5 | 2.1±1.2 | 65 | 2.6±1.1 | 1.8±1.3 | 21 |
| CO_2_ (ppm) | 579±143 | 598±153 | 62 | 532±100 | 496±59 | 19 |
| Temp (ºC) | 22.6±2.9 | 23.0±3.0 | 63 | 20.7±4.2 | 20.1±3.9 | 20 |
| RH (%) | 48±15 | 47±13 | 63 | 46±22 | 47±19 | 20 |
| ^1^ Number of measurements  ^2^ Mean ± standard deviation  ^3^ No measurement | | | | | | |
